# Social Determinants of Health Patterns in Children with Severe Disease Due to SARS-CoV-2 Infection—An Exploratory Approach

**DOI:** 10.3390/children12111515

**Published:** 2025-11-09

**Authors:** Joshua Prabhu, Sebastian Acosta, Fabio Savorgnan, Ananth V. Annapragada, Usha Sethuraman

**Affiliations:** 1 Division of Emergency Medicine, Department of Pediatrics, Children’s Hospital of Michigan, 3901 Beaubien Blvd, Detroit, MI 48201, USA; prabh1j@cmich.edu (J.P.); sethu1u@cmich.edu (U.S.); 2 Pediatrics–Critical Care, Baylor College of Medicine, One Baylor Plaza, Houston, TX 77030, USA; fabio.savorgnan@bcm.edu; 3 Texas Children’s Hospital-Radiology, Baylor College of Medicine, One Baylor Plaza, Houston, TX 77030, USA; 4 Carmen and Ann Adams Department of Pediatrics, Central Michigan University, 1200 S Franklin St, Mount Pleasant, MI 48859, USA

**Keywords:** COVID-19, social determinants of health, disease severity, Emergency Medicine, pediatrics, children, SARS-CoV-2

## Abstract

**Highlights:**

**What are the main findings?**

- Families with children with SARS-CoV-2 infection reported a high level of adverse social determinants of health. Two adverse social determinants of health patterns (caregiver use of drugs/alcohol and social discrimination/lack of support) were associated with severe pediatric COVID-19.

**What are the implications of the main findings?**

- Combinations of adverse social determinants of health in children may have a higher impact on disease severity than in isolation. Our results can inform future studies and the development of tailored interventions to address these patterns of social risk factors.

**Abstract:**

**Background/Objectives:** Research on the association of adverse social determinants of health (SDOH) with severe pediatric coronavirus disease (COVID-19) is limited. We examined associations between SDOH patterns and COVID-19 severity in children. **Methods:** We conducted a prospective, observational study of children (<18 years) with symptomatic SARS-CoV-2 infection evaluated in an urban pediatric emergency department (March 2021–April 2022) in Detroit, Michigan. Caregivers completed a 34-item survey based on the Healthy People 2030 framework. Severe disease was defined as the occurrence of respiratory/cardiac failure or death within four weeks of diagnosis. Continuous and categorical variables were described using medians and percentages, respectively. Associations between disease severity and risk factors were determined using chi-square tests. Association rule mining was used for feature selection, followed by multivariate logistic regression. **Results:** We analyzed data from 354 children [6–12 years: 31.1%, Female: 51.1%, Black: 59%, not Hispanic: 84.7%, public insurance: 77.1%, chronic condition: 27.4%]. Of the total, 113 children had severe disease. Most caregivers were 30–44 years old (53.1%), had less than a college degree (70.4%), and income < USD 50,000 (75.2%). Adverse SDOH reported included food/housing insecurity (24.6%), no support (64.7%), unmet childcare needs (35.9%), and lack of transportation (12.7%). After controlling for age, sex, medical history, income, and obesity, severe disease was associated with caregiver use of drugs/alcohol (OR:5.92, *p* < 0.001) and social discrimination/lack of support (OR: 1.74, *p* = 0.030). **Conclusions:** Two SDOH patterns (caregiver use of drugs/alcohol and social discrimination/lack of support) were associated with severe COVID-19. Further studies are needed to confirm findings and develop interventions.

## 1. Introduction

The Coronavirus 2019 (COVID-19) pandemic has highlighted the significant impact of social determinants of health (SDOH) on disease severity and outcomes. Studies in adults both in the United States and globally have shown that factors such as socioeconomic status, race, urban versus rural location, and access to healthcare are strongly associated with the incidence and severity of COVID-19 [1,2,3]. Individuals from lower socioeconomic backgrounds and racial minorities have experienced higher rates of hospitalization and mortality [2]. Factors like housing instability, lack of food, and unemployment have worsened the impact of COVID-19 on vulnerable populations [3]. For example, families with public insurance rather than private insurance were more likely to face disproportionate burdens of the COVID-19 pandemic and were more likely to have worse access to care for children with asthma during the pandemic [4]. Sharma et al. described the compounding impact of the pandemic on households with children across multiple health needs [5].

However, information on the impact of SDOH on COVID-19 disease severity in children is limited. Javalkar et al. reported that higher social vulnerability index (SVI), lower socio-economic status, Hispanic ethnicity and Black race were associated with the development of Multi-System Inflammatory Syndrome in Children (MISC), a severe form of COVID-19 [6]. Another study on a cohort of Latin American children with MIS-C reported that food insecurity, higher distance from a health center, not possessing a private vehicle to transport the patient to hospital and having a home in poor condition were associated with low left ventricular ejection fraction, shock and need for respiratory support, and negatively affected their outcomes [7]. A study from Brazil described the spatial distribution of COVID-19 related deaths in children and the impact of regional inequities, health disparities and poverty on poor outcomes [8]. However, most of these studies have used indices such as social vulnerability index and area deprivation index (ADI), which arbitrarily select SDOH variables and may not measure individual-level child health directly. A recent report suggests that patterns of SDOH may be more important determinants of health outcomes in children [9]. This cohort study included data from a diverse sample of children across 21 sites in the United States (rural, urban and mountainous). Significant disparities in child developmental health outcomes were observed across four patterns of SDOH: (1) affluence, (2) high-stigma environment, defined as the highest implicit bias and discrimination toward women and sexual and gender minority groups, (3) high socioeconomic deprivation, and (4) high crime and drug sale rates coupled with lower education and densely populated areas with the worst health outcomes found with pattern 3. Our group previously found that no specific SDOH was associated with severe COVID-19 in children [10]. However, the impact of a combination of SDOH factors on severe disease in children is unknown and requires further investigation. Our objective was to identify patterns of SDOH that may be associated with severe COVID-19 in children. 

## 2. Materials and Methods

**Figure 1:**
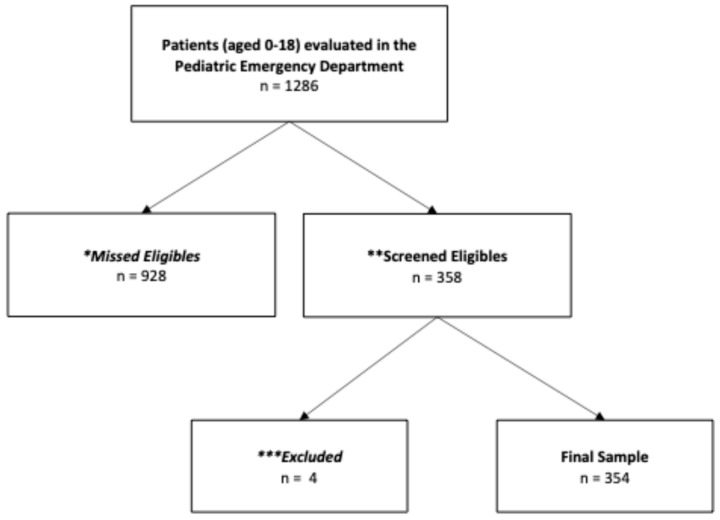
Study Flowchart. * Missed Eligibles: Study team was not available to survey and enroll patients. ** Screened Eligibles: Study team was available to survey and enroll patients. *** Excluded: No legal guardian was present or legal guardian refused survey.

*Study design and location*: This was a prospective, observational study of a convenience sample of 354 children (severe disease, n = 113) evaluated in the emergency department (ED) of an urban tertiary care children’s hospital between March 2021 and April 2022 (Figure 1). The ED is a level one trauma center with > 80,000 annual ED visits. 

## 3. Enrollment and Exclusion

*Population*: Children < 18 years who were diagnosed with severe acute respiratory syndrome coronavirus 2 (SARS-CoV-2) infection [via positive Retro Transcriptase Polymerase Chain Reaction (RT PCR) test, serology, or epidemiological link] between March 2021 and April 2022 in an urban pediatric emergency department in Detroit, Michigan, were enrolled. Written informed consent and, where required, an assent were obtained from all legal guardians. The study was conducted in accordance with the Declaration of Helsinki and approved by the Institutional Review Board of University of Pittsburgh (MOD21010046-003, approval date: 25 February 2021).

*Exclusion criteria*: Presentation during times when the study team was not available, those unaccompanied by a legal guardian or biological parent and refusal of consent. Since this study was a part of a larger study, named Severity Predictors Integrating Salivary TranScriptomics and proteomics with Multi neural network Intelligence in SARS-CoV2 infection in Children (SPITS MISC), which evaluated the impact of the infection on salivary levels of microRNAs, children with head, oral or dental trauma, seizures and pregnancy were also excluded as these conditions may alter the expression of salivary microRNAs [11,12,13].

*Survey*: Once informed consent was obtained, the participant’s parent completed an electronic 34-item survey. The survey questions were developed based on the Healthy People 2030 framework and covered five key domains (Table 1). Specifically, all social determinant variables were derived from standardized items based on the *Healthy People 2030* framework and the *PhenX Toolkit* for SDOH [14,15,16,17]. Each domain and variable was operationalized in a 34-question survey as follows.

Unmet childcare needs were evaluated through a direct caregiver-reported item asking whether, in the past month, the family was unable to obtain childcare when it was really needed (Yes/No).

Unmet social needs were identified through questions regarding inability to access essential household resources (e.g., clothing, utilities, phone, internet, childcare, school supplies).

Community context was assessed through two Likert-scale items measuring *neighborhood collective efficacy* (“People in this neighborhood help each other out”) and *perceived neighborhood safety* (“Children are safe in our neighborhood”). Responses were dichotomized as *strongly agree* versus *not strongly agree*.

Social support was measured through items assessing both emotional and tangible support (e.g., “I have people I can turn to for help with my problems”, “I have someone who will listen when I need to talk”) using a 5-point frequency scale ranging from *never* to *always*.

Perceived discrimination was assessed through a multi-item scale that asked participants how often they experienced disrespect, poor service, or harassment, and follow-up questions identifying the perceived reason (e.g., race, gender, income, or other factors).

Caregiver drug/alcohol use was assessed using the CAGE-AID screening questions, a validated instrument for identifying potential substance use problems [18].

All items were self-reported and dichotomized or categorized according to established scoring guidelines and prior studies.

These variables were then analyzed under the five domains of the *Healthy People 2030* SDOH framework: Economic Stability, Education Access and Quality, Social and Community Context, Healthcare Access and Quality, and Neighborhood and Built Environment. One survey per family was included in the analysis. Therefore, in families with multiple siblings who were enrolled into the study, only the oldest child’s survey was included.

*Exposures*: The five key domains included Economic Stability (household income, pandemic-related income/employment loss, food insecurity, housing stability), Education Access and Quality (caregiver education level), Social and Community Context (caregiver social support, unmet social needs, unmet childcare needs), Healthcare Access and Quality (unmet healthcare needs, health insurance status), and Neighborhood and Built Environment (neighborhood collective efficacy, neighborhood perceived safety, transportation needs).

### 3.1. Data Collected

The following data were collected from the medical chart of the child: age, sex, race, ethnicity, insurance type (public/private/self payor), SARS-CoV-2 status, weight, height, past medical history, symptoms and signs at presentation, admitted to hospital (yes/no), laboratory results if any, treatments given and outcome (discharge/death). Severity classification (severe/non-severe) was assigned by the study team based on a priori definition. The following data were collected from the parent survey: age, sex, race, ethnicity and the five key domains of exposures previously stated. All data were entered into a REDCap [(v 11.4.4), hosted at Wayne State University, Detroit, MI, USA. All data were collected and entered by a trained research assistant and then verified by a second study team member.

### 3.2. Definitions

*SARS-CoV-2 infection*: The diagnosis of SARS-CoV-2 infection for this study was made if any of the following were present: (a) positive PCR test, (b) positive serology or (c) epidemiology link (participant had symptoms and signs of SARS-CoV-2 infection and close household contact with a family member who was positive for the infection in the previous two weeks). This was per the Center of Disease Control’s definition at the time of the study [19].

*Chronic medical conditions were defined* as having at least one of the following conditions: asthma, cancer, cardiovascular disease, chronic kidney disease, chronic lung disease, diabetes, hypertension, an immunosuppressive condition, serious mental disease such as mental health disorders including schizophrenia, neurodevelopmental disorders, major depression, acute psychosis, and mood disorders, or sickle cell disease.

*Severe disease was defined* as including at least one of the following: requirement of high flow oxygen > 8 L; bilevel or continuous positive airway pressure or noninvasive positive pressure ventilation; treatment with inotropes, mechanical ventilation, extracorporeal membrane oxygenation; or occurrence of cardiac arrest or death within four weeks of diagnosis.

### 3.3. Statistical Analysis

Continuous variables were described using medians and interquartile ranges. Categorical variables were described with frequencies and percentages. The association between disease severity and each of the categorical risk factors were determined using the chi-square test. These tests were implemented using “scipy” package version 1.9.1. Since the number of risk factors included in the study was large in relation to the sample size for a multivariate analysis, we used association rule mining (ARM) as a feature selection technique to identify risk factors with the strongest association with disease severity. ARM is a data mining method that explores logic-based relationships, called association rules, between antecedent and consequent variables in a dataset [20]. These rules, expressed as “if-then” statements, reveal how frequently certain variables or groups of variables are found together in the dataset. The strength of the association is quantified using three metrics: support, confidence, and lift. Thresholds for these three metrics are employed to deem an association sufficiently strong. Support is the probability with which antecedent and consequent variables occur together in the set, i.e., an “if and only if” rule. Confidence is the probability that the consequence will be found provided that the antecedent has been found, i.e., an “if-then” rule. Lift is a measure of how much the presence of the antecedent increases the probability of the consequence occurring, relative to the whole dataset. We defined the rules in which severe disease was the consequence, and then explored the strength of risk factors as antecedents. We set a minimum support of 10%, minimum confidence of 20% and a minimum lift of 1.00 as the thresholds. The risk factors that satisfied these conditions after the ARM was applied were subsequently included in a multivariate logistic regression for severe versus non-severe disease. An association (either from the chi-square test or the logistic regression) was considered statistically significant for a *p*-value < 0.05. The ARM method was implemented using “mlxtend” package version 0.23.4. Logistic regression was implemented using the “statsmodels” package version 0.13.2. All the analyses were coded in Python platform version 3.8.3. 

## 4. Results

A total of 354 children with SARS-CoV-2 infection (severe disease, n = 113) were included in the study. Demographic characteristics of the total sample and stratification by COVID-19 severity (non-severe vs. severe) are noted in Table 2. Regarding child characteristics, age ranges were as follows: 0–1 years (n = 43, 15.0%), 2–5 years (n = 78, 27.3%), 6–12 years old (n = 89, 31.1%), and 13–17 years (n = 76, 26.6%). The majority of the children were female (n = 181, 51.1%), Black (n = 209, 59.0%), not Hispanic (n = 300, 84.7%), and had public insurance (n = 273, 77.1%). More than one-fourth of the children (n = 97, 27.4%) had a chronic medical condition. Caregiver demographics showed that the majority were aged 30–44 years (n = 188, 53.1%), had less than a college degree (n = 249, 70.4%), and reported an annual household income of <USD 75,000 (n = 266, 75.2%). 

The most commonly reported adverse SDOH factors reported included having no emotional or tangible support (n = 229, 64.7%), unmet social or childcare needs (n = 127, 35.9%), lack of neighborhood help (n = 125, 35.3%), food insecurity (n = 87, 24.6%) and lack of neighborhood safety (n = 76, 21.5%). There were differences between the two groups (severe vs. non-severe disease) in the child’s age (*p *= 0.037), sex (*p *= 0.034), race (*p *= 0.016), body weight (*p *= 0.008), and presence of a chronic condition (*p *= 0.029). Caregiver age (*p *= 0.002), education (*p *= 0.022) and household income (*p *= 0.022) also differed between the two groups.

ARM was performed, and identified several factors and patterns associated with severe illness (Table 3). These included child’s age (adolescent/teenage) (support = 0.14, confidence = 0.38, lift = 1.21), child body weight (obese) (support = 0.11, confidence = 0.41, lift = 1.30), family medical history (support = 0.23, confidence = 0.37, lift = 1.15), patient chronic condition (support = 0.17, confidence = 0.35, lift = 1.11), social discrimination/lack of support (support = 0.12, confidence = 0.47, lift = 1.46), and caregiver use of drug/alcohol (support = 0.18, confidence = 0.38, lift = 1.18). 

These features, identified by the ARM method, were then included as variables in a multivariable logistic regression analysis, while also augmenting the list of explanatory variables with insurance status (private vs. others) and household income (USD 25 k or more vs. less) as socio-economic features (Table 4). The following SDOH factors remained significantly associated with severe COVID-19 outcomes: caregiver use of drugs/alcohol (OR = 5.92, *p *= 0.001) and social discrimination/lack of support (OR = 1.74, *p *= 0.030). This was after controlling for other factors including age of patient [teenager] (OR = 1.25, *p *= 0.392), sex [male] (OR = 1.54, *p *= 0.077), obesity (OR = 4.59, *p *= 0.040), previous family medical history (OR = 1.77, *p *= 0.034), patient chronic conditions (OR = 1.04, *p *= 0.883), insurance status [private] (OR = 1.17, *p *= 0.620), and household income [USD 25 k or more] (OR = 1.46, *p *= 0.149).

## 5. Discussion

This study investigated the complex interplay between SDOH and the severity of COVID-19 in a cohort of children evaluated in an urban tertiary care ED. We found two patterns of SDOH, including caregiver use of drugs/alcohols and the presence of social discrimination/lack of social support, that were associated with severe COVID-19 after adjusting for known risk factors. To our knowledge, this is the first study that has evaluated the impact of combinations of adverse SDOH on the severity of COVID-19 in children. Our findings align with the growing understanding that health outcomes are not solely determined by individual risk factors, but are profoundly influenced by the broader social and environmental contexts in which individuals live, work and play [21].

Consistent with previous findings in adult populations, we found that caregiver age and education level, household income, family medical history, and the child’s race, sex, age and weight were associated with COVID-19 severity in children. The strong association between child obesity and severe COVID-19 likely reflects underlying physiological mechanisms, such as chronic inflammation and impaired immune responses, that increase susceptibility to severe illness [22,23]. Similarly, family medical history may indicate a genetic predisposition or shared environmental exposures within households that contribute to more severe disease trajectories.

We found that caregiver use of drugs/alcohol was associated with increased odds of severe disease in the child. Previous studies have noted the deleterious and enduring effects of parental substance use on a child’s health and development [24,25,26]. It has been reported that children who grow up in households with parental substance use have a higher risk of developing hyperactivity, depression, injuries, and other mental and behavioral disorders [27,28,29,30]. The significant association with caregiver drug/alcohol use, often intertwined with economic instability, mental health challenges, and lack of social support, can impact a caregiver’s ability to provide consistent care, access healthcare services, and maintain a stable home environment, all of which can indirectly influence a child’s health and vulnerability to severe infection [31]. This highlights the interconnectedness of caregiver well-being and child health outcomes within the context of SDOH. 

The association between social discrimination/lack of support and severe COVID-19 underscores the impact of systemic inequities and social isolation on health. Discrimination can lead to chronic stress, reduced access to resources, and diminished trust in healthcare systems. Lack of social support can limit access to practical assistance, emotional coping resources, and information, all of which are crucial during a health crisis [32]. These factors can create a vulnerability that exacerbates the impact of infection. While our study, like others, identified demographic disparities in COVID-19 severity, framing the issue solely by race or income risks oversimplification. Our findings suggest that patterns of social and environmental challenges, which may disproportionately affect certain demographic groups, may be critically associated with severe outcomes. 

This study has several strengths. It is the first study to explore the impact of patterns of adverse SDOH on disease severity due to SARS-CoV-2 infection in a large cohort of children. The SDOH data were collected prospectively based on the *Healthy People 2030 framework* and *PhenX* Toolkit framework, thus reducing recall bias while ensuring rigor and comparability across studies. The use of the novel ARM approach captures the complex interplay among social and environmental influences on child health. The identification of specific critical patterns provides actionable targets for screening and future interventions in pediatric and emergency care settings.

This study has several limitations. First, as a single-center study, our findings may not be generalizable to other hospitals and broader outpatient populations, particularly those with a higher proportion of mild disease presentations. A previous study has shown the impact of blood type on the severity of COVID-19 [33]. Since we did not collect this information, it may have impacted our results. We also did not include questions regarding caretaker mental health, which may be associated with drug/alcohol use, in our analysis. Additionally, the study lacked a control group of children without SARS-CoV-2 infection, limiting our ability to distinguish virus-specific effects from baseline pediatric health variations. The inclusion criteria were restricted to children with confirmed SARS-CoV-2 infection, which may have introduced selection bias. Furthermore, recruitment occurred between March 2021 and April 2022, a period during which the Delta variant initially predominated, followed by the Omicron variant. This temporal shift in circulating variants may have influenced disease severity and symptomatology, affecting the interpretation of our results [34,35]. In the event of multiple siblings that were enrolled in the study, we only included the oldest child’s survey response in the analysis, which may have biased our results. However, since all the enrolled siblings belonged to the same family with similar SDOH, this bias is unlikely. Also, the reliance on caregiver self-report for SDOH data may be subject to recall bias or social desirability bias. Variables such as social support, perceived discrimination, and neighborhood safety reflect subjective perceptions, which may be influenced by caregiver stress, mood, or cultural expectations. This may introduce reporting or perception bias, particularly in domains like community context and unmet childcare needs, where personal experience plays a significant role. Additionally, we did not include objective community-level indicators (e.g., census-derived poverty rates, crime data, or neighborhood deprivation indices), which could have validated or complemented subjective measures. Despite these limitations, using caregiver-reported data provides valuable insight into lived experiences and psychosocial stressors that objective indices may miss. Future studies should employ mixed-methods designs combining validated self-report tools with objective neighborhood-level data and possibly qualitative interviews to minimize bias and enrich our understanding of how SDOH influences pediatric disease severity.

Additionally, while ARM identified relevant patterns, the complexity of SDOH means that other important combinations may exist that were not captured or analyzed. Lastly, as an observational study using cross-sectional data, our analysis cannot establish causality between SARS-CoV-2 infection and clinical outcomes.

Future studies with larger, multicenter cohorts, longitudinal designs, and more comprehensive, objective measures of SDOH are needed to validate these findings and explore the underlying mechanisms. Acknowledging and addressing the combined impact of these social and environmental factors is crucial for developing effective public health strategies and ensuring equitable healthcare access for vulnerable pediatric populations.

## 6. Conclusions

In conclusion, this study provides compelling evidence that two patterns of SDOH, caregiver drug/alcohol use and social discrimination/lack of support, were associated with increased odds of severe COVID-19 in children. Our study underscores the complex interplay of social and environmental contexts and disease outcomes. Further studies are required to confirm these findings so that targeted interventions may be developed to mitigate health disparities and improve health outcomes for children.

## Figures and Tables

**Table 1:** SDOH Measures and Definitions.

SDOH Framework	Variables
Economic Stability	Household income: Low, middle, high Pandemic-related income/employment loss: Yes/no Food insecurity: 2-item screener Housing stability: 2-item screener
Education Access and Quality	Caregiver education level: Categorical (from less than 9th grade through graduate or professional degree)
Social and Community Context	Caregiver social support: Emotional and tangible support, yes/no Unmet social needs: Yes/no Unmet childcare needs: Yes/no
Healthcare Access and Quality	Unmet healthcare needs (any medical, dental, vision, or mental health service): Yes/no Health insurance status: Public, private, or no insurance
Neighborhood and Built Environment	Neighborhood collective efficacy: Strongly agree vs. not Neighborhood perceived safety: Strong agree vs. not Transportation needs: Yes/no

**Table 2:** Demographic characteristics of study sample, total and by pediatric COVID-19 severity. *p*-values correspond to the Chi-square test.

Characteristic N (%)	Total N = 354	Non-Severe N = 241	Severe N = 113	*p*-Value
Caregiver Age				**0.002**
18–29 years	116 (32.8)	93 (38.6)	23 (20.4)	
30–44 years	188 (53.1)	122 (50.6)	66 (58.4)	
45+ years	33 (9.3)	16 (6.6)	17 (15.0)	
Unknown	17 (4.8)	10 (4.1)	7 (6.2)	
Child Race				**0.016**
Black	209 (59.0)	152 (63.1)	57 (50.4)	
White	86 (24.3)	47 (19.5)	39 (34.5)	
Asian	10 (2.8)	8 (3.3)	2 (1.8)	
American/Alaska Native	1 (0.3)	0 (0.0)	1 (0.9)	
Other Race/Unknown	48 (13.6)	34 (14.1)	14 (12.4)	
Child Ethnicity				0.997
Hispanic	38 (10.7	26 (10.8)	12 (10.6)	
Not Hispanic	300 (84.7)	204 (84.6)	96 (85.0)	
Unknown	16 (4.5)	11 (4.6)	5 (4.4)	
Child Sex				**0.034**
Male	173 (48.9)	108 (44.8)	65 (57.5)	
Female	181(51.1)	133 (55.2)	48 (42.5)	
Child Body Weight				**0.008**
Obese	87 (24.6)	51 (21.2)	36 (31.9)	
Overweight	40 (11.3)	33 (13.7)	7 (6.2)	
Normal	199 (56.2)	132 (54.8)	67 (59.3)	
Underweight	22 (6.2)	19 (7.9)	3 (2.7)	
Unknown	6 (1.7)	6 (2.5)	0 (0.0)	
Child age (yrs)				**0.037**
0–1	43 (15.0)	30 (15.6)	13 (13.8)	
2–5	78 (27.3)	58 (30.2)	20 (21.3)	
6–12	89 (31.1)	63 (32.8)	26 (27.7)	
13–18	76 (26.6)	41 (21.4)	35 (37.2)	
Economic Stability				
Household income				**0.022**
Low (USD 0 k–USD 25 k)	162 (45.8)	123 (51.0)	39 (34.5)	
Mid (USD 25 k–USD 75 k)	104 (29.4)	67 (27.8)	37 (32.7)	
High (USD 75 k or more)	45 (12.7)	25 (10.4)	20 (17.7)	
Unknown	43 (12.1)	26 (10.8)	17 (15.0)	
Housing instability				0.965
Yes	70 (19.8)	47 (19.5)	23 (20.4)	
No	284 (80.2)	194 (80.5)	90 (79.6)	
Food insecurity				0.750
Yes	87 (24.6)	62 (25.7)	25 (22.1)	
No	241 (68.1)	162 (67.2)	79 (69.9)	
Unknown	26 (7.3)	17 (7.1)	9 (8.0)	
Child chronic condition ^1^				**0.029**
Yes	97 (27.4)	57 (23.7)	40 (35.4)	
No	257 (72.6)	184 (76.3)	73 (64.6)	
Education				
Caregiver highest degree of education				**0.022**
<High school	47 (13.3)	35 (14.5)	12 (10.6)	
High school graduate/GED	202 (57.1)	144 (59.8)	58 (51.3)	
University/College degrees	74 (20.9)	48 (19.9)	26 (23.0)	
Unknown	31 (8.8)	14 (5.8)	17 (15.0)	
Social and Community Context				
Lack of emotional support	108 (30.5)	76 (31.5)	32 (28.3)	0.625
Lack of tangible support	121 (34.2)	88 (36.5)	33 (29.2)	0.218
Unmet social needs	79 (22.3)	55 (22.8)	24 (21.2)	0.844
Unmet childcare needs	48 (13.6)	36 (14.9)	12 (10.6)	0.347
Healthcare Access and Quality				
Unmet health needs				0.574
Yes	21 (5.9)	16 (6.6)	5 (4.5)	
No	332 (94.1)	225 (93.4)	107 (95.5)	
Insurance status				0.083
Public (Medicaid, CHIP)	273 (77.1)	192 (79.7)	81 (71.7)	
Private (Employer-based)	71 (20.1)	45 (18.7)	26 (23.0)	
No insurance	10 (2.8)	4 (1.7)	6 (5.3)	
Neighborhood and Built Environment				
Lack of Transportation				0.327
Yes	45 (12.7)	34 (14.1)	11 (9.7)	
No	309 (87.3)	207 (85.9)	102 (90.3)	
Neighborhood help				0.567
Definitely/Somewhat agree	229 (64.7)	153 (63.5)	76 (67.3)	
Definitely/Somewhat disagree	125 (35.3)	88 (36.5)	37 (32.7)	
Neighborhood safety				0.368
Definitely/Somewhat agree	278 (78.5)	193 (80.1)	85 (75.2)	
Definitely/Somewhat disagree	76 (21.5)	48 (19.9)	28 (24.8)	

^1^. Chronic conditions include a diagnosis of asthma, diabetes, hypertension, cardiovascular disease, chronic lung disease, chronic kidney disease, immunosuppressive conditions, or sickle cell disease.

**Table 3:** Association rules mining to select features associated with severe illness. Sample size N = 354. The thresholds for selection of features are a minimum support of 0.10, a minimum confidence of 0.20 and a minimum lift of 1.00.

Pattern	Support	Confidence	Lift
Adolescent/teenager	0.14	0.38	1.21
Body weight (obese)	0.11	0.41	1.30
Family medical history	0.23	0.37	1.15
Patient chronic condition	0.17	0.35	1.11
Social discrimination/lack of support	0.12	0.47	1.46
Caregiver use of drug/alcohol	0.18	0.38	1.18

**Table 4:** Association rule mining informed multivariable logistic regression results presenting adjusted odds ratios and *p*-values for the associations between SDOHs and severe vs. non-severe outcomes. Sample N = 354. DF residuals = 341. DF model = 12. LLR *p*-value = 8.06 × 10^−7^.

	Coefficient	Odds Ratio	*p*-Value
Intercept	−1.674		** 0.001 **
C (Age) [teenager vs. other]	0.221	1.25	0.392
C (Sex) [male vs. female]	0.429	1.54	0.077
C (Body weight type) [obese vs. other]	1.524	4.59	** 0.040 **
C (Caregiver use of drug/alcohol) [yes vs. no]	1.779	5.92	** 0.001 **
C (Family medical history) [any vs. none]	0.570	1.77	** 0.034 **
C (Social discrimination/lack of support) [yes vs. no]	0.554	1.74	** 0.030 **
C (Patient chronic condition) [any vs. none]	0.037	1.04	0.883
C (Insurance) [private vs. others]	0.156	1.17	0.620
C (Household income) [USD 25 k or more vs. other)	0.376	1.46	0.149

## Data Availability

The original data presented in the study are available with approval at https://www.ncbi.nlm.nih.gov/projects/gap/cgi-bin/study.cgi?study_id=phs002549.v1.p1 (accessed on 9 October 2025).

## Abbreviations

COVID-19—Coronavirus 2019; SDOH—Social Determinants of Health; IQR—Interquartile Range; SVI—Social Vulnerability Index; MISC—Multi System Inflammatory Syndrome in Children; ADI—Area Deprivation Index; ED—Emergency Department; SARS-CoV-2—Severe Acute Respiratory Syndrome Coronavirus 2; RT PCR—Retro Transcriptase Polymerase Chain; SPITS MISC—Severity Predictors Integrating Salivary TranScriptomics and proteomics with Multi neural network Intelligence in SARS-CoV2 infection in Children; ARM—Association Rule Mining; OR—Odds Ratio.
